# Aryl-Capped Lysine-Dehydroamino Acid Dipeptide Supergelators as Potential Drug Release Systems

**DOI:** 10.3390/ijms231911811

**Published:** 2022-10-05

**Authors:** Carlos B. P. Oliveira, Renato B. Pereira, David M. Pereira, Loic Hilliou, Tarsila G. Castro, José A. Martins, Peter J. Jervis, Paula M. T. Ferreira

**Affiliations:** 1 Chemistry Centre, School of Sciences, University of Minho, 4710-057 Braga, Portugal; 2 REQUIMTE/LAQV, Laboratório de Farmacognosia, Departamento de Química, Faculdade de Farmácia, Universidade do Porto, R. Jorge Viterbo Ferreira, n 228, 4050-313 Porto, Portugal; 3 Institute for Polymers and Composites, University of Minho, 4800-058 Guimarães, Portugal; 4 Center of Biological Engineering, University of Minho, Campus de Gualtar, 4710-057 Braga, Portugal; 5 LABBELS—Associate Laboratory, 4800-122 Guimarães, Portugal

**Keywords:** dehydrodipeptides, lysine, self-assembly, supramolecular hydrogels, critical gelation concentration, drug delivery systems

## Abstract

Employing amino acids and peptides as molecular building blocks provides unique opportunities for generating supramolecular hydrogels, owing to their inherent biological origin, bioactivity, biocompatibility, and biodegradability. However, they can suffer from proteolytic degradation. Short peptides (<8 amino acids) attached to an aromatic capping group are particularly attractive alternatives for minimalistic low molecular weight hydrogelators. Peptides with low critical gelation concentrations (CGCs) are especially desirable, as the low weight percentage required for gelation makes them more cost-effective and reduces toxicity. In this work, three dehydrodipeptides were studied for their self-assembly properties. The results showed that all three dehydrodipeptides can form self-standing hydrogels with very low critical gelation concentrations (0.05–0.20 wt%) using a pH trigger. Hydrogels of all three dehydrodipeptides were characterised by scanning tunnelling emission microscopy (STEM), rheology, fluorescence spectroscopy, and circular dichroism (CD) spectroscopy. Molecular modelling was performed to probe the structural patterns and interactions. The cytotoxicity of the new compounds was tested using human keratinocytes (HaCaT cell line). In general, the results suggest that all three compounds are non-cytotoxic, although one of the peptides shows a small impact on cell viability. In sustained release assays, the effect of the charge of the model drug compounds on the rate of cargo release from the hydrogel network was evaluated. The hydrogels provide a sustained release of methyl orange (anionic) and ciprofloxacin (neutral), while methylene blue (cationic) was retained by the network.

## 1. Introduction

Hierarchical self-assembly is ubiquitous in nature, playing an essential structural role within cellular scaffolds [1]. The benefits of these molecular assemblies can be harnessed in the laboratory to produce materials with specific properties and functionalities [2]. In the last decades, the self-assembly of peptides to afford supramolecular hydrogels has been heavily explored for a variety of biomedical applications, such as in sustained drug delivery, materials for 3D-cell culture, tissue engineering, wound healing, biosensing, bioprinting, and many others [3,4,5,6,7,8]. Short peptides (<8 amino acids) capped with an aromatic group on the *N*-terminus are particularly attractive to researchers, as these hydrogelators have the advantages of inherent biocompatibility, ease of synthesis, tuneable mechanical properties, the ability to respond to external stimuli, and close mimicry of the extracellular matrix [9]. The gelation process is usually initiated by decreasing the solubility of a peptide in aqueous solution through an external trigger, which can be achieved through a temperature change (cooling), a pH change (addition of acid to anionic solutions), a solvent switch, or chemically through the action of an enzyme or light [10,11,12,13,14]. Such a trigger allows the formation of the required intramolecular non-covalent interactions, such as hydrogen bonds, π–π stacking interactions, van der Waals forces, and hydrophobic interactions [15]. These interactions combine to allow the formation of fibrils and fibres, which are able to trap water during the aggregation process [16,17].

**Figure 1:**
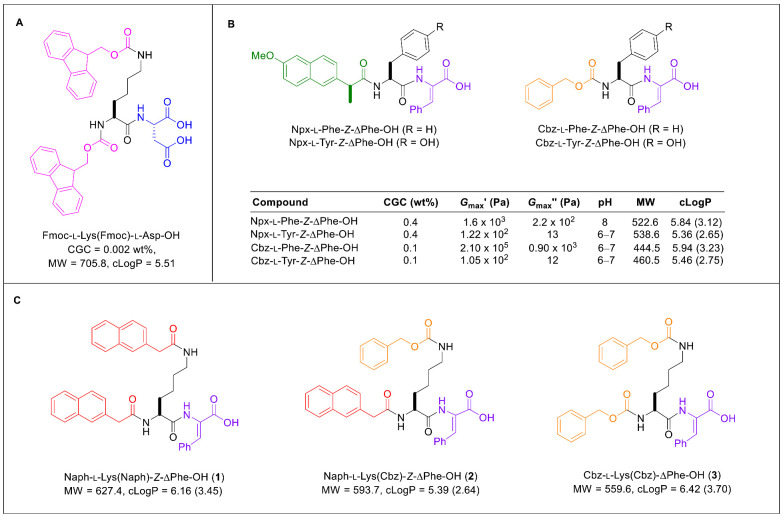
(**A**) Structure of Gazit’s dipeptide ‘ultragelator’. (**B**) A summary of the gelation properties of our previously studied dehydrodipeptides. (**C**) The structures of the dehydrodipeptides **1**–**3** to be studied in this work. cLogP value were obtained using https//molinspiration.com, accessed on 9 May 2022 (the values in parentheses refer to deprotonated carboxylate salt form).

There is a continuous search to identify the simplest building blocks and to optimise the critical gelation concentration [18]. Small peptide hydrogelators with low critical gelation concentration (CGC) are especially sought, as using a lower quantity of peptide reduces any putative toxic effect and reduces the time, cost, and effort involved in peptide preparation [19]. Typically, the reported CGCs of peptide hydrogels are in the range of 0.1–1.0 wt%; however, some notable examples where the CGCs are much lower have been reported in the literature. Wang et al. reported a hexadecapeptide hydrogelator with a CGC value of 0.007 wt% [20], while Tian et al. have reported that a short peptide hydrogelator (Nap–GFFY–OMe) exhibits a CGC value of 0.01 wt% [21]. Recently, the group of Gazit reported a minimalist dipeptide with the lowest CGC ever reported, forming hydrogels at concentrations as low as 0.002 wt% (28.3 × 10^−6^ M) [22]. Remarkably, this corresponds to a ratio of over one million water molecules for every peptide molecule. The structure of this ultragelator consists of a lysine amino acid residue coupled to aspartic acid, where the two amine groups of lysine are capped with fluorenylmethoxycarbonyl (Fmoc) groups (Figure 1A).

Our research group has studied dehydropeptides *N*-capped with aromatic groups, which form hydrogels with favourable rheological properties for drug delivery applications [23,24,25,26,27,28]. Furthermore, triggered drug delivery was achieved with composite dehydropeptide-based hydrogels incorporating magnetic and plasmonic nanoparticles, under external non-invasive activation with benign optical-magnetic stimuli [29]. Some examples of our previously studied dehydrodipeptides, along with a summary of their gelation properties, are shown in Figure 1B.

Herein, we describe the synthesis and characterisation of three hydrogelators (**1**–**3**) based on dehydroamino acids (Figure 1C) that are able to provide hydrogels at the lowest critical gelation concentrations ever described for dehydropeptide hydrogels. Guided by the best previous examples, a lysine residue was incorporated into the structure [22]. As Fmoc groups are known to have toxic degradation products, gelators were designed where the Fmoc groups of Gazit’s hypergelator were replaced by carboxybenzyl (Cbz) and/or 2-naphthylacetyl (Naph) groups [30,31]. Switching the base-labile Fmoc group for more stable Cbz and Naph groups would also allow a pH change to be employed as the gelation trigger. The aspartic acid residue was replaced by a dehydrophenylalanine residue. We have previously shown that the presence of a dehydroaminoacid residue increases the proteolytic resistance of a hydrogel [18]. In addition, dehydroamino acid residues are also known to aid the gelation process through the restriction of conformational freedom [23].

## 2. Results and Discussion

### 2.1. Synthesis

**Scheme 1:**
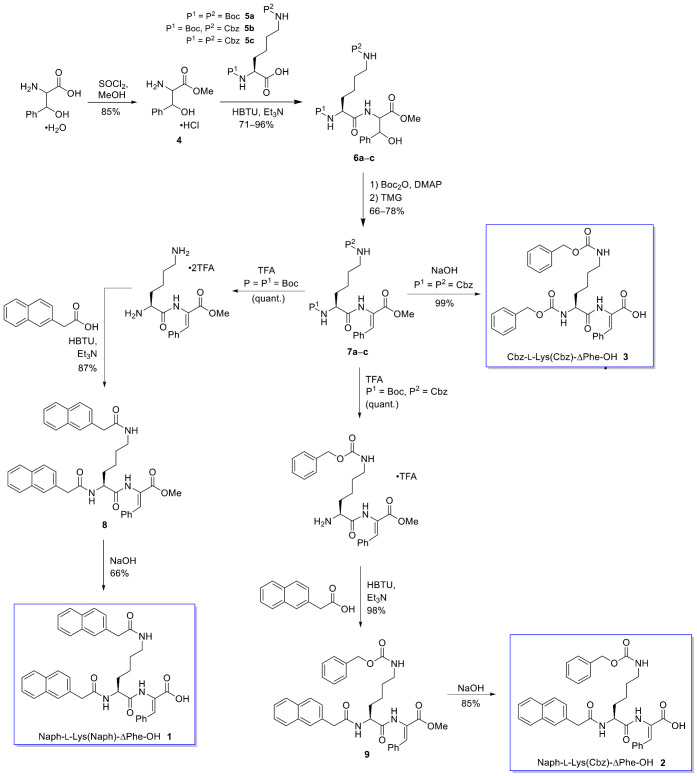
Synthesis of lysine-containing dehydrodipeptides **1**–**3**.

The dehydrodipeptides **1**–**3** were synthesised using conventional solution-phase peptide synthesis (Scheme 1) [29]. Starting from β-hydroxyphenylalanine [H-D,L-Phe(β-OH)-OH•H_2_O], an initial esterification using thionyl chloride in methanol afforded the corresponding methyl ester **4**. A subsequent amide coupling reaction with diprotected lysine derivatives **5a**–**c** in the presence of 2-(1H-benzotriazole-1-yl)-1,1,3,3-tetramethylaminium hexafluorophosphate (HBTU) afforded the corresponding dipeptides **6a**–**c** as diastereomeric mixtures. The β-hydroxycarbonyl moiety was dehydrated by treatment with di-tert-butyl dicarbonate (Boc_2_O) in the presence of 4-dimethylaminopyridine (DMAP), followed by *N*,*N*,*N’*,*N’*-tetramethylguanidine (TMG), to afford the protected dehydrodipeptides **7a**–**c**. Two stereogenic centres are removed in this step, and therefore the initial diastereomeric mixtures were converged to single enantiomers (**7a**–**c**). A deprotection step of **7a** was performed with trifluoroacetic acid to remove both of the tert-butoxy-carbonyl (Boc) groups, followed by a coupling reaction with two equivalents of 2-naphthylacetic acid, in the presence of HBTU, to afford compound **8**. Compound **9** was accessed in a similar way from peptide **7b**; in this case there was only one Boc to remove, and one Naph group to be coupled. Compound **7c** already contained the required Cbz groups attached the nitrogen functionalities of the lysine residue. Final hydrolysis reactions of **8**, **9**, and **7c** using NaOH proceeded uneventfully to afford compounds **1**–**3**, respectively (Scheme 1).

### 2.2. Gelation Study

**Figure 2:**
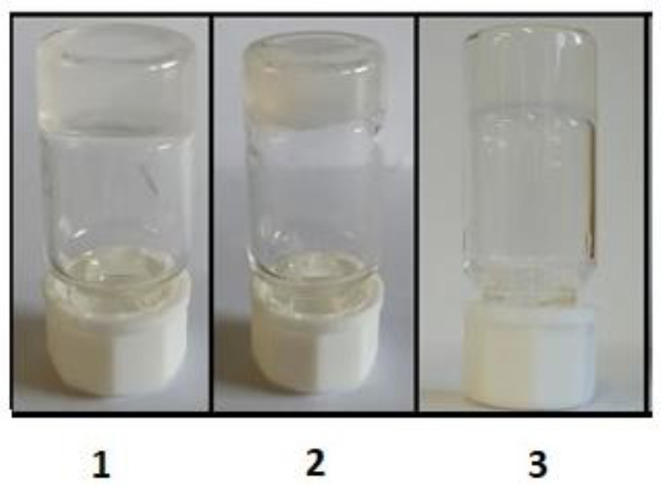
Optical images of hydrogels formed by dehydrodipeptides **1**–**3**.

As previously mentioned, low molecular weight hydrogelators are able to self-assemble due to noncovalent interactions, which allow monomeric building blocks to self-associate into ordered fibrous structures, which later entangle and interact with each other to form the 3D hydrogel network [15,16,17]. Several methods have been described for triggering gelation, such as pH change, heating–cooling cycles, enzymatic catalysis, the addition of chelating metal ions, and sonification [10,11,12,13,14]. The synthesised peptides **1**–**3** were only sparingly soluble in buffer solutions in the physiological pH range (6.0–8.0). However, they could be dissolved in water upon pH adjustment to pH 10 with the addition of sodium hydroxide (1.0 M). Hydrogelation was then triggered by the slow decrease in pH achieved through the addition of D-glucono-δ-lactone (GdL), which slowly hydrolyses to D-gluconic acid. Hydrogelation using GdL has been shown to be superior to the addition of mineral acids such as HCl, since hydrolysis to D-gluconic acid is slower than the rate of diffusion, leading to more uniform hydrogels [11]. Using these conditions, peptides **1**–**3** produced self-standing hydrogels (Figure 2). The critical gelation concentration (CGC) of compounds **1**–**3** was assessed by varying the peptide concentrations and conducting vial inversion tests (Table 1).

The CGC values obtained for peptides **1**–**3** are significantly lower than the usual CGCs reported for low molecular weight peptide-based supramolecular hydrogels. However, the CGCs for peptides **1**–**3** are still significantly higher than those reported for Fmoc-L-Lys(Fmoc)-Asp-OH (the peptide with the lowest known CGC) [22]. The peptide Fmoc-L-Lys(Fmoc)-Asp-OH has two carboxylic acid moieties at the *C*-terminus that compensate for the hydrophobicity arising from the two Fmoc groups. In our case, dehydrodipeptides **1**–**3** have a hydrophobic aromatic dehydrophenylalanine residue instead of aspartic acid, resulting in a significantly more hydrophobic structure. The higher CGC of dehydrodipeptides **1**–**3** may also be explained by considering the gelation methodology used. The hydrogels of compounds **1**–**3** were prepared using a pH trigger, whereas the hydrogel of the dipeptide Fmoc-L-Lys(Fmoc)-Asp-OH was prepared using a solvent switch method. As previously described, the CGC depends on the method used to trigger gelation [10]. Employing a solvent switch method with compounds **1**–**3** failed to produce hydrogels. Conversely, the hydrogel Fmoc-L-Lys(Fmoc)-Asp-OH is unsuitable for gelation using a pH change, as the Fmoc groups are base-labile. Therefore, a direct comparison of the gelators under identical gelation conditions was not possible.

### 2.3. Scanning Transmission Electron Microscopy (STEM) Studies

**Figure 3:**
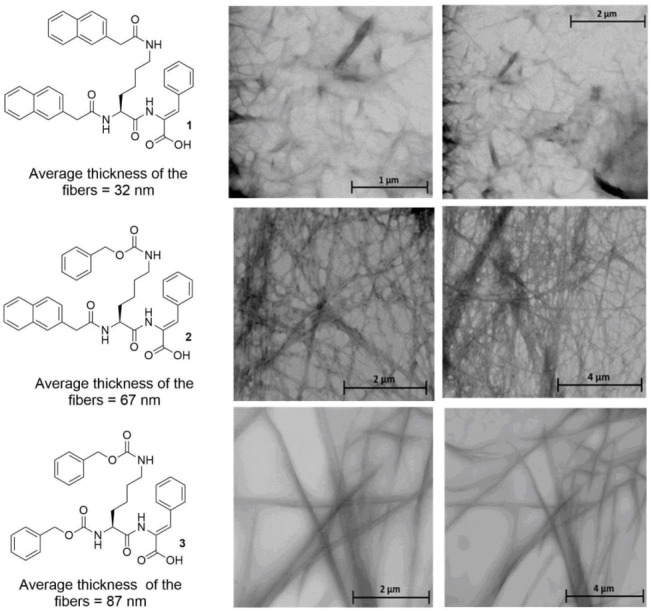
Scanning transmission electron microscopy (STEM) images of hydrogels **1**–**3** at 0.20 wt%.

The nanostructures of hydrogels **1**–**3** were studied using scanning transmission electron microscopy (STEM). The STEM images displayed networks of fibres assembled into the 3D networks of the hydrogels (Figure 3). From Figure 3, it is possible to conclude that there is a tendency for hydrogels to form with thicker fibres when the dehydropeptide contains carboxybenzyl capping groups, compared to those containing naphthalene protecting groups. This is an interesting observation, suggesting that (depending on the end application) the thickness of the fibres can be tuned by varying the protecting groups used.

### 2.4. Circular Dichroism (CD) Spectroscopy

**Figure 4:**
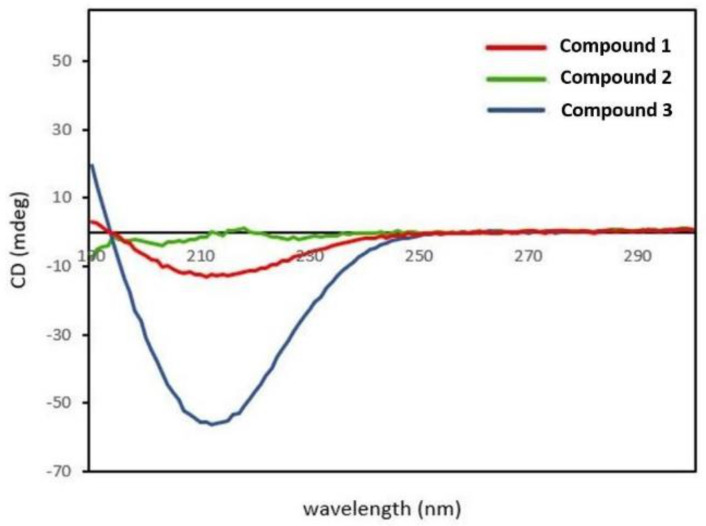
CD spectra of aqueous solutions of compounds **1**–**3** (0.01 wt%).

The circular dichroism (CD) spectra of hydrogelators **1**–**3** are presented in Figure 4. To avoid the light scattering effects which can occur in turbid gels, CD spectra were acquired with hydrogelator solutions of lower concentrations than the CGC. In the preparation of the samples to be analysed by CD, D-glucono-δ-lactone (GDL) was added to basic solutions of the hydrogelator solutions to simulate the experimental conditions of gel formation. The CD spectra of dehydrodipeptides **1** and **3** are very similar, displaying negative bands at 215 nm and 213 nm, respectively (Figure 4). These results suggest the presence of a β-sheet aggregation pattern. The CD spectrum of dehydrodipeptide **2** suggests a predominance of random coils due to the two small negative bands at 202 nm and 228 nm. The only structural difference between hydrogelator **2** and hydrogelators **1** and **3** is the presence of different *N*-capping groups. Thus, the data suggest that the *N*-capping groups on lysine have a significant effect on the self-assembly process.

### 2.5. Rheological Studies

Rheological studies provide structural information about the type, number, and stiffness of the overall network responsible for hydrogelation. Consequently, rheology is an important tool used for characterising supramolecular hydrogels [32].

**Figure 5:**
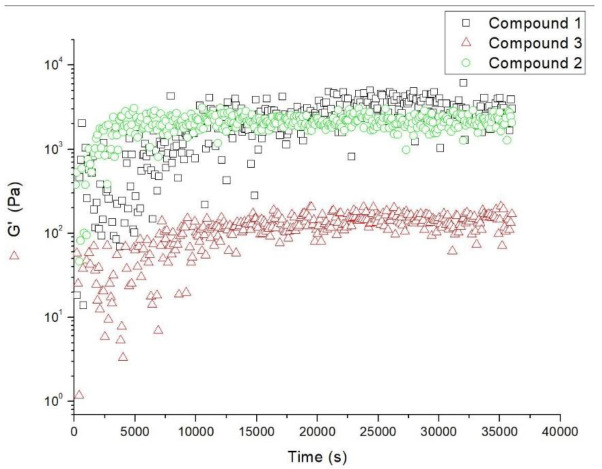
Elastic modulus during the kinetic process of gelation for dehydrodipeptides 1–3 at 0.2 wt%.

The gelation kinetics of dehydrotripeptides **1**–**3** are presented in Figure 5. From the results obtained, dehydrodipeptide **2** had the fastest gelation kinetics, providing a gel in around 20 min. Conversely, the hydrogels prepared from dehydrodipeptides **1** and **3** displayed gelation times of 2.8 h and 2.6 h, respectively. These time values were inferred from the time taken to reach a time-independent G’ value.

**Figure 6:**
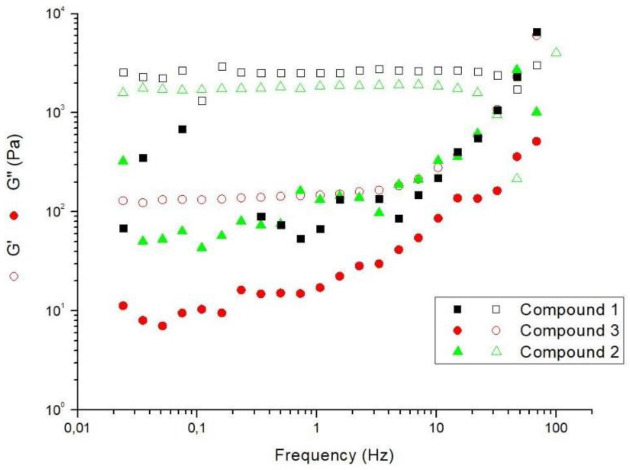
Frequency dependence of the shear elastic G’ and G″ module for the dehydrodipeptides **1**–**3** at 0.20 wt%.

Upon reaching the structural equilibrium established from the G’ and G’’ values versus time, a frequency-sweep from 100 Hz down to 0.1 Hz was performed, with an applied strain of 0.01%, to afford the mechanical spectra displayed in Figure 6. All three hydrogels prepared from dehydrodipeptides **1**–**3** showed a G’ essentially constant over the frequency domain tested, whereas G’’ displays a stronger frequency dependence.

In general, the mechanical spectra of the three hydrogels are similar, suggesting that the elastic network responsible for the hydrogels mechanical response share structural similarities. As expected, the G’ is higher than G″ for all three hydrogels (Table 2). By comparing the G’ values of hydrogels obtained from dehydrodipeptides **1**–**3**, it can be observed that hydrogels **1** and **2** are the stiffest and most elastic, while hydrogel **3** was observed to be the least elastic for the gelling conditions used in Figure 6. In addition, hydrogels **1** and **2** show G’ values similar to those obtained for other hydrogelators obtained from dehydrodipeptides in our research group, at similar concentrations and pH values [24,25,26,27,28,29]. The value of G’ of hydrogel 3 is rather low, which could be a potential disadvantage for using this specific hydrogel for sustained drug release.

**Figure 7:**
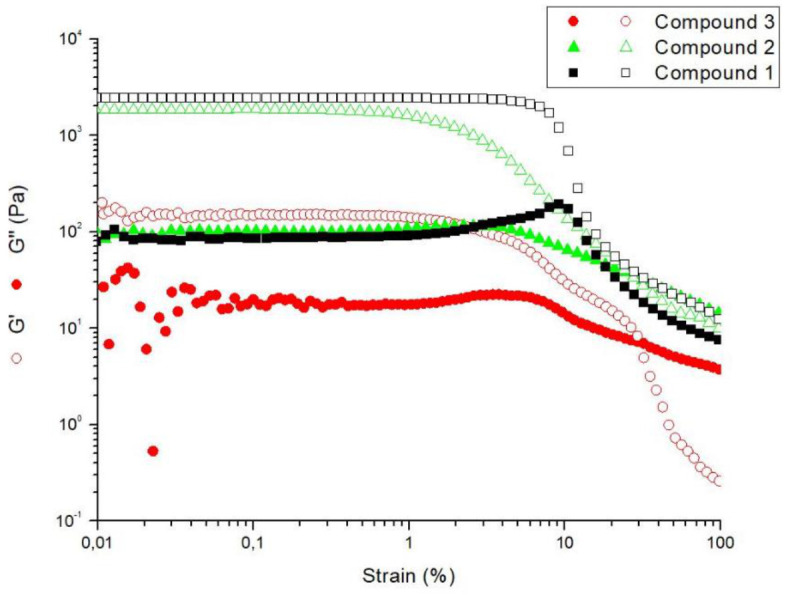
Strain dependence of the shear elastic G’ and loss G’’ moduli for dehydrodipeptides **1**–**3** at 0.20 wt%.

Following the frequency sweep, the hydrogels of **1**–**3** were submitted to a strain sweep where the frequency was maintained at 1 Hz (Figure 7). Hydrogels of dehydrodipeptides **1**, **2**, and **3** break up at strains of 100%, 73.3%, and 35%, respectively. Interestingly, the hydrogel prepared from peptide **1** was both the most elastic and strain resistant among the three dehydrodipeptides hydrogels. On the other hand, the hydrogel formed from **3** was the least elastic and the weakest, while the hydrogel formed from **2** showed a balance between the other two hydrogels, both in elasticity and strength. These data suggest an influence of the aromatic capping groups. Overall, dehydrodipeptides *N*-protected with Naph groups produced hydrogels of superior rheological properties to the dehydropeptides *N*-protected with Cbz groups.

### 2.6. Biocompatibility and Cytotoxicity Studies

**Figure 8:**
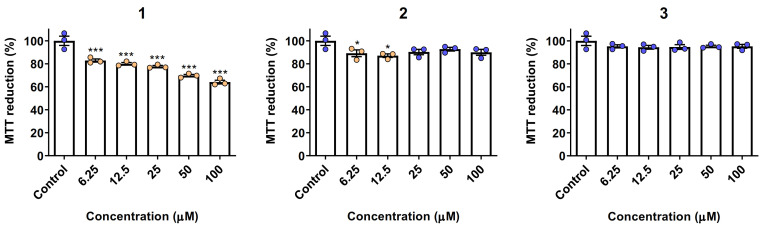
Viability of HaCaT cells treated with **1**–**3** for 24 h at the concentrations presented. * *p* < 0.05, *** *p* < 0.001.

Peptides **1***–***3** were initially evaluated for their potential impact on the viability of human keratinocytes, namely the HaCaT cell line (Figure 8). None of the three dehydrodipeptides showed a critical loss of cell viability, suggesting that the peptides are suitable for further studies related to biological applications. However, the results also show that dehydrodipeptide **1** has an impact on cell viability at higher concentrations, reaching a maximum of 30% loss at 100 μM. Peptides **2** and **3** were mostly devoid of any effect on viability, even at the highest concentrations tested.

**Figure 9:**
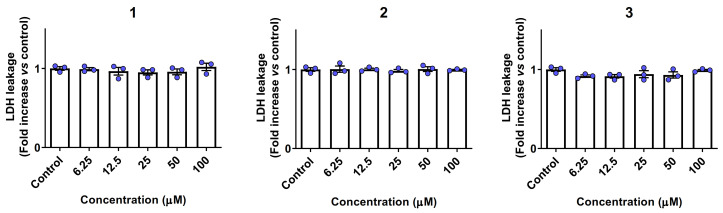
LDH activity found in the culture media of HaCaT cells treated with **1**–**3** for 24 h at the concentrations presented. Triton X-100 was used as a positive control to lyse cells.

Due to the small effect of peptide **1** cell viability, we again evaluated if the molecules elicit necrosis in treated HaCaT cells. To this end, a lactate dehydrogenase (LDH) leakage assay was performed (Figure 9) in order to evaluate the level of plasma membrane damage (an increase in the leakage of cytosolic LDH is a typical hallmark of necrosis). No statistically significant increase in LDH levels was detected versus control in concentrations up to 100 µM, suggesting that the compounds do not elicit necrosis. Triton X-100 at 1% was used to completely lyse the cells, causing a 5-fold increase in LDH leakage versus control (data not shown).

Overall, we can conclude that peptides **1***–***3** are not cytotoxic, although peptide **1** has a small impact on cell viability, which does not translate into loss of membrane integrity.

### 2.7. Drug Delivery Study

**Figure 10:**
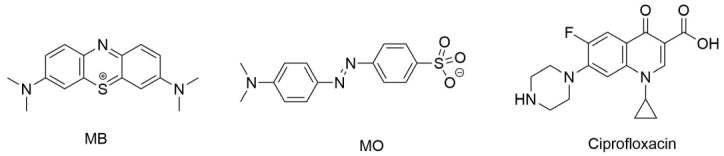
Small molecule cargos for release: Methylene blue (MB), methyl orange (MO), and ciprofloxacin.

Due to the intrinsic characteristics of supramolecular hydrogels, these compounds have the potential to be used as drug delivery systems able to overcome the pharmacokinetics limitations of certain drugs, such as poor aqueous solubility or short half-lives in vivo. The hydrogel prepared from dehydrodipeptide **1** was investigated for its capacity to entrap, and then release, model compounds. To this effect, two dyes and an antibiotic were incorporated in the hydrogel matrix. Thus, methylene blue (MB), methyl orange (MO), and ciprofloxacin were chosen as cationic, anionic, and overall neutral cargo (Figure 10). The release of each cargo molecule from the two hydrogels was assessed.

Hydrogels of peptide **1** containing model compounds were prepared using the same conditions described in Section 4.2, but with the water component being replaced by methylene blue solution, methyl orange solution, or ciprofloxacin solution. In a slightly modified version of the method described by Abraham et al. [33], a solution of simulated body fluid (SBF) was layered on top of the hydrogel surface and then the percentage of the compound release was recorded versus time. This analysis was carried out by UV-vis spectroscopy for the dyes, and by HPLC for ciprofloxacin.

**Figure 11:**
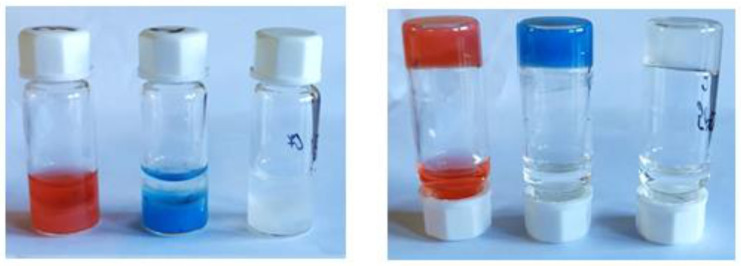
Representative images of hydrogels obtained from dehydrodipeptide **1** loaded with MO (left), MB (centre), and ciprofloxacin (right) layered with SBF solution (1.5 mL) following a saturating release study. After 168 h, 37% of MO and 23% of ciprofloxacin were released from the hydrogel.

**Figure 12:**
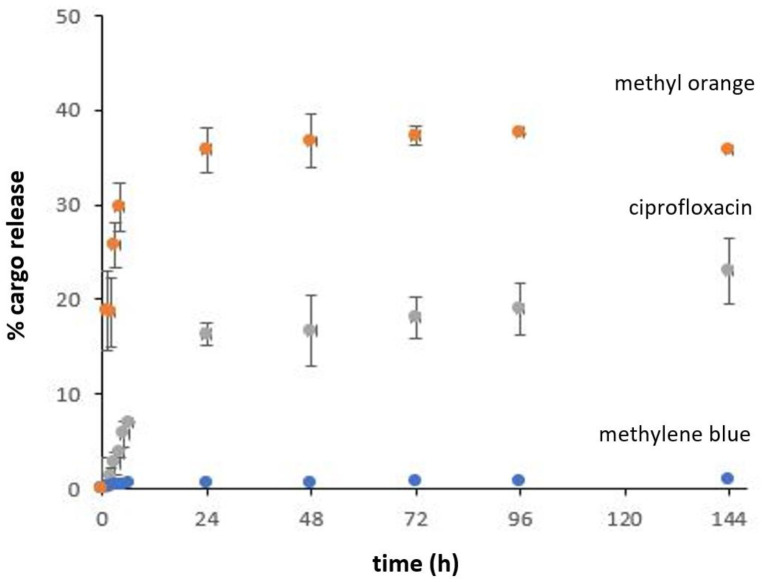
Percentage release of methylene blue, methyl orange, and ciprofloxacin from hydrogel **1** over time.

In assays studying the release of cationic MB from hydrogels of peptide **1**, the top layer remained colourless and transparent over 7 days, suggesting that only a small amount of MB was released from the hydrogel matrix (Figure 11). This result was confirmed by UV-vis spectroscopy, which revealed that only 2% the cationic MB was released by the hydrogel network. The results obtained for MO-containing hydrogels showed that this compound was more readily released by the network of hydrogel **1** (Figure 12), with 37% of the cargo being released after 7 days, although a plateau was reached after 24 h. In the case of ciprofloxacin, it was found that hydrogel **1** released 23% of this drug cargo after 7 days. As was the case with MO, most of the cargo was released in the first 24 h (Figure 12).

To quantitatively evaluate the drug release from the hydrogel **1**, several mathematical models were tested to see which one fitted the tested hydrogel. The Korsmeyer–Peppas model was the preferred model for polymeric systems. Korsmeyer described a simple relationship which described drug release from a polymeric system that includes both diffusion and erosion of the polymer chain [34].

$$\frac{Mt}{M}=Kk^{n}$$

### 2.8. Molecular Modelling Studies

**Figure 13:**
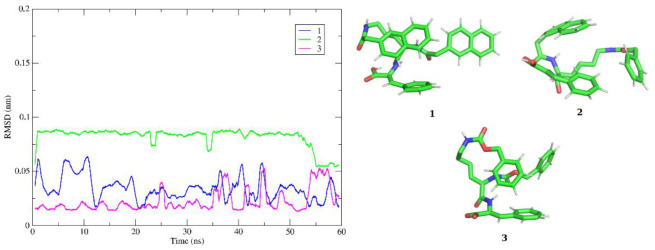
Left: Root mean square deviation (RMSD) of peptides backbone using backbone fitting, along simulation time. Right: Most typical conformation obtained for peptides **1**–**3** from cluster analysis. Carbons are represented in green, nitrogen in blue, oxygen in red, and polar/aromatics hydrogens in white. Peptides in united-atom stick representation.

Molecular dynamics (MD) simulations were carried out to unveil the structural preference and interactions of dehydrodipeptides **1**–**3** when the *N*-capping group and/or the lysine side chain are varied. First, the peptide monomers in infinite dilution conditions were tracked to infer their intramolecular interactions and conformational populations. The peptides backbone root mean square deviation (RMSD) were analysed, and a clustering method resulted in the most likely conformations (Figure 13). The overall behaviour is to approximate the aromatic moieties in all three cases, suggesting that *π*-stacking interactions take place even intramolecularly. The RMSD curves show that the backbone is very stable, presenting low values of deviation from the generated structure/topology calculated in ATB server (quantum mechanical calculations). Consequently, only one cluster was found for each 60 ns of peptide simulation, indicating one representative conformation that minimises the RMSD variance when fitted against all others.

**Figure 14:**
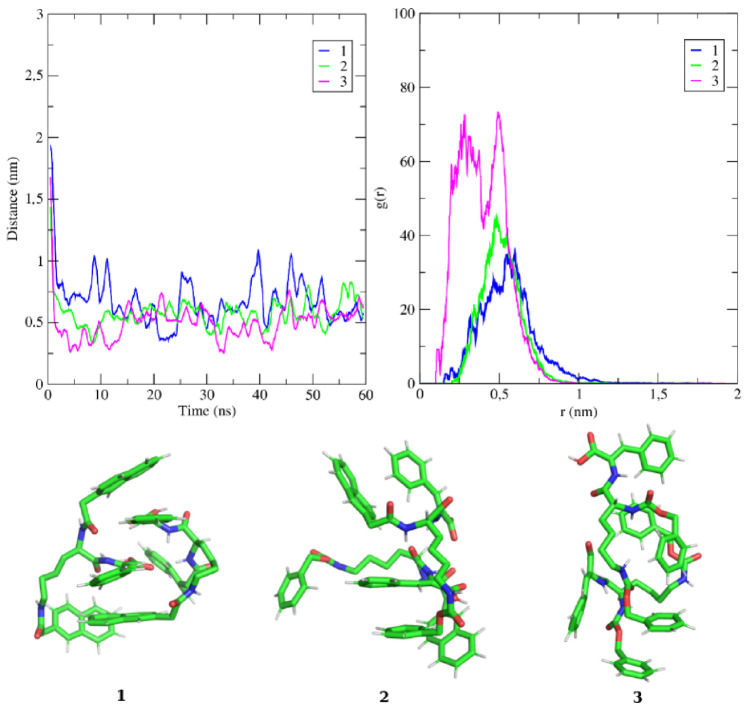
Centre of mass (COM) intermolecular distance between dehydrodipeptide units along simulation time; COM radial distribution functions (RDFs) of the peptides’ interaction probability density vs. distance and peptide dimers aggregation snapshots (**1**–**3**). The colour scheme follows the description in Figure 13.

The aggregation phenomenon was also studied by MD simulations. In this case, two peptide units were placed in the simulation box with an average distance from each other above 2 nm. Visual inspection of the trajectories demonstrated that the peptides approached readily and remained close in most of the 60 ns simulation windows. Additionally, the centre-of-mass (COM) intermolecular distances and the radial distribution functions (RDFs) between the two peptide molecules were measured as an aggregation metric, indicating that the peptides are bound when a distance up to the intermolecular distance cut-off (of 1.4 nm) is observed. Figure 14 shows the intermolecular distance, the RDFs, and a representative aggregated structure for each case.

The RDFs indicate how the probability density of a particle varies as a function of the distance from the other. In Figure 14, it should be noted that the dehydrodipeptides of system assembled from **3** are able to interact at shorter distances, probably due to the more elongated conformation of the peptide units. Importantly, this same structural pattern could be responsible for facilitating the *β*-sheet interactions and the thicker fibres observed in CD and STEM analysis. Furthermore, the smaller capping groups of **3** might facilitate the process. The sharper RDF curve for **3** indicates a great propensity for self-assembly; however, the other two systems also present large populations under 1 nm distance, which also suggests a facile aggregation process.

For each of the dipeptides **1**–**3**, varied types of *π*−*π* interactions are observed: Sandwich, t-shape, and parallel displaced. Notably, this type of interaction occurs both inter- and intramolecularly. The snapshots in Figure 14 show examples of multiple and diverse *π*-interactions. The flexible carbon tails of lysine allow the carboxybenzyl or naphthalene groups to interact with the *N*-capping group or with ΔPhe. Thus, all aromatic groups actively participate in self-assembly.

The presence of intermolecular hydrogen bonds was also tracked along the simulation time for peptides **1**–**3**. Although hydrogen bonding collectively contributes to the self-assembly process under the infinite dilution conditions, this interaction is less important than the *π*−*π* interactions.

## 3. Conclusions

In this work we describe the synthesis, characterisation, and gelation properties of three new supergelators **1**–**3**, containing a lysine residue *N*-capped with different aro-matic moieties and a dehydrophenylalanine residue. Peptides **1**–**3** produced hydrogels with very low critical gelation concentration values of 0.05, 0.07, and 0.20 wt%, the lowest CGCs reported for dehydropeptide hydrogelators. The CGC values are an order of magnitude higher than the lowest CGC reported by Gazit for the ultragelator, Fmoc-L-Lys(Fmoc)-Asp-OH. However, our new hydrogelators present some structural advantages. Switching the Fmoc capping groups for carboxybenzyl or naphthalene capping groups increases the structural stability of the compounds (particularly under basic conditions) and allows alternative gelation methods to be employed. Furthermore, these new structures also avoid the toxicity issues associated with the metabolic degradation of Fmoc groups [30,31].

MD simulations disclosed the interaction patterns through inter- and intramolecular π−π interactions and revealed that dipeptide **3** adopts a more elongated conformation. Dipeptide **3** has a larger propensity for the aggregation into dimers on short time-scales, through π−π interactions. However, it is feasible that these dimers have less affinity for aggregation with each other in higher-ordered structures. Therefore, a higher peptide concentration might be required for the aggregates to form β-sheets and span the gelled volume, thus accounting for the higher CGC of dipeptide **3**.

The rheological properties of the new hydrogels displayed a storage modulus (G′) significantly higher than the loss modulus (G″), which confirmed a viscoelastic behaviour characteristic of supramolecular hydrogels, and that the elastic range these hydrogels was close to was that observed in biological tissues. STEM analysis revealed that all hydrogels display fibrillar structures. The biocompatibility of these peptides was evaluated by cytotoxicity assays. The peptides were initially evaluated for their potential impact on the viability of human keratinocytes (HaCaT cell line). Generally, the results suggest that the peptides are not cytotoxic, despite compound **1** showing a small impact on cell viability. Finally, in sustained release assays, the effects of the charge present on model drug compounds on the rate of cargo release from the hydrogel networks were studied using cationic (methylene blue), anionic (methyl orange), and neutral cargo (ciprofloxacin). Generally, the hydrogels retained methylene blue inside the hydrogel network, while the release rate of methyl orange and ciprofloxacin was much faster.

Overall, the new compounds developed can produce hydrogels in very low concentrations, are biocompatible, and are efficient drug delivery systems for anionic and neutral drugs.

## 4. Materials and Methods

### 4.1. Synthesis

Compounds **1**–**3** were prepared by synthetic methodologies developed in our laboratory and fully characterised by ^1^H and ^13^C NMR spectroscopy (NMR) and High-Resolution Mass Spectrometry (HRM). Experimental procedures and characterisation data for compounds **4**, **5a**, **6a**–**c**, **7a**–**c**, **8**, and **9** are presented in the Supplementary Materials. Spectra were acquired on a Bruker Avance III 400 spectrometer, operating at 400.13 MHz and 100.62 MHz, for ^1^H and ^13^C NMR, respectively. HRMS data were acquired from the mass spectrometry service of the University of Vigo, Spain. The partition coefficient between water and n-octanol (log P) of compounds **1**–**3** was calculated using Molinspiration Cheminformatics software Molinspiration, Slovensky Grob, Slovak Republic, 2017 (https://www.molinspiration.com, accessed on 9 May 2022). The calculated log P is a sum of fragment-based contributions and correction factors, and it is used as a quantitative descriptor of compound lipophilicity.

#### 4.1.1. Synthesis of Naph-L-Lys(Naph)-Z-ΔPhe-OH (**1**)

Naph-L-Lys(Naph)-*Z*-ΔPhe-OMe (**8**) was dissolved in 1,4-dioxane (12.4 mL), and a solution of 1.0 M NaOH (1.6 equiv, 3.7 mL, 1.00 mmol) was added. The reaction was followed by TLC until no starting material was detected (typically about 4 h). The organic solvent was removed under reduced pressure, and the reaction mixture was acidified to pH 2–3 with KHSO4 (1.0 M). The solid was collected by filtration and then washed with Et_2_O. The solid was identified as Naph-L-Lys(Naph)-*Z*-ΔPhe-OH (**1**) (0.247g, 66%). ^1^H NMR (400 MHz, DMSO-d_6_) δ: 1.29–1.49 (4H, m, γ-CH_2_ and δ-CH_2_ of Lys), 1.54–1.81 (2H, m, β-CH_2_ of Lys), 2.91–3.12 (2H, m, ε-CH_2_ of Lys), 3.55 (2H, s, 1 × CH_2_ of Naph), 3.66 (2H, s, 1 × CH_2_ of Naph), 4.39 (1H, m, α-CH of Lys), 7.23–7.30 (3H, m, ArH), 7.39–7.51 (6H, m, ArH and β-CH of ΔPhe), 7.57–7.64 (2H, m, ArH), 7.70–7.89 (9H, m, ArH), 8.07 (1H, t, J 4.8 Hz, 1 × NH), 8.35 (1H, d, J 8.0 Hz, 1 × NH), 9.56 (1H, s, NH of ΔPhe), 12.62 (1H, br, s, CO_2_H of ΔPhe). ^13^C NMR (100.6 MHz, DMSO-d_6_, δ): 22.7 (CH_2_, γ-CH_2_ of Lys), 28.8 (CH_2_, δ-CH_2_ of Lys); 31.3 (CH_2_, β-CH_2_ of Lys); 38.6 (CH_2_, ε-CH_2_ of Lys); 42.1 (CH_2_, 1 × CH_2_ of Naph), 42.6 (CH_2_, 1 × CH_2_ of Naph), 52.6 (CH, α-CH of Lys), 125.52 (CH, Ar), 125.53 (CH, Ar), 126.0 (CH, Ar), 126.1 (CH, Ar), 126.6 (CH, Ar), 127.2 (CH, Ar), 127.3 (CH, Ar), 127.40 (CH, Ar), 127.41 (CH, Ar), 127.50 (CH, Ar), 127.57 (CH, Ar), 127.6 (CH, Ar), 127.7 (CH, Ar), 128.4 (CH, Ar), 129.1 (CH, Ar), 130.0 (CH, Ar), 131.8 (C, Ar), 131.9 (CH, β-CH of ΔPhe), 133.0 (C, α-C of ΔPhe), 133.5 (C, Ar), 134.1 (C, Ar), 134.2 (C, Ar), 166.2 (C, C=O), 169.9 (C, C=O of Naph), 170.2 (C, C=O of Nah), 171.6 (C, C=O). HRMS (ESI) *m/z*: [M + H]^+^ calcd for C_39_H_38_N_3_O_5_ 628.2811; found: 628.2820.

#### 4.1.2. Synthesis of Naph-L-Lys(Cbz)-Z-ΔPhe-OH (**2**)

Naph-L-Lys(Cbz)-*Z*-ΔPhe-OMe (**9**) was dissolved in 1,4-dioxane (11 mL) and a solution of 1.0 M NaOH (1.6 equiv, 3.2 mL, 1.50 mmol) was added. The reaction was followed by TLC until no starting material was detected (typically about 4 h). The organic solvent was removed under reduced pressure, and the reaction mixture was acidified to pH 2–3 with KHSO4 (1.0 M). The solid was collected by filtration and then washed with Et2O. The solid was identified as Naph-L-Lys(Cbz)-*Z*-ΔPhe-OH (**2**) (0.474g, 85%). ^1^H NMR (400 MHz, DMSO-d_6_)δ: 1.21–1.42 (4H, m, γ-CH_2_ and δ-CH_2_ of Lys), 1.55–1.80 (2H, m, β-CH_2_ of Lys), 2.79-2.96 (2H, m, ε-CH_2_ of Lys), 3.64 (1H, d, J 14.0 Hz, C*H*_A_H_B_ of Naph), 3.69 (1H, d, J 14.0 Hz, CH_A_C*H*_B_ of Naph), 4.40 (1H, dd, J 13.2 Hz, 8.4 Hz, α-CH_2_ of Lys), 4.99 (2H, s, CH_2_ of Cbz), 7.18–7.50 (10H, m, ArH and β-CH of ΔPhe and 1 × N*H*), 7.56–7.67 (3H, m, ArH), 7.61–7.89 (6H, m, ArH), 8.34 (1H, d, J 8.0 Hz, NH), 9.54 (1H, s, NH of ΔPhe). ^13^C NMR (100.6 MHz, DMSO-d_6_, δ): 22.6 (CH_2_, γ-CH_2_ of Lys), 29.2 (CH_2_, δ-CH_2_ of Lys), 31.4 (CH_2_, β-CH_2_ of Lys), 40.2 (CH_2_, ε-CH_2_ of Lys), 42.1 (CH_2_, CH_2_ of Naph), 52.7 (CH, α-CH of Lys), 65.1 (CH_2_, CH_2_ of Cbz), 125.5 (CH, Ar), 126.01 (CH, Ar), 126.04 (C, Ar), 126.6 (CH, Ar), 127.3 (CH, Ar), 127.40 (CH, Ar), 127.46 (CH, Ar), 127.5 (CH, Ar), 127.6 (CH, Ar), 127.7 (CH, Ar), 128.35 (CH, Ar), 128.39 (CH, Ar), 129.1 (CH, Ar), 129.9 (CH, Ar), 131.7 (C, Ar), 131.8 (CH, β-CH of ΔPhe), 132.9 (C, α-C of ΔPhe), 133.6 (C, Ar), 134.2 (C, Ar), 137.2 (C, Ar), 156.1 (C, C=O), 166.2 (C, C=O), 170.1 (C, C=O), 171.6 (C, C=O). HRMS (ESI) *m/z*: [M + H]^+^ calcd for C_35_H_36_N_3_O_6_ 594.2604; found: 594.2580.

#### 4.1.3. Synthesis of Cbz-L-Lys(Cbz)-Z-ΔPhe-OH (**3**)

Cbz-L-Lys(Cbz)-*Z*-ΔPhe-OMe (**7c**) (774 mg, 1.35 mmol) was dissolved in 1,4-dioxane (13.5 mL) and a solution of 1.0 M NaOH (1.5 equiv, 2.02 mL, 2.02 mmol) was added. The reaction was followed by TLC until no starting material was detected (typically about 4 h). The organic solvent was removed under reduced pressure, and the reaction mixture was acidified to pH 2–3 with KHSO_4_ (1.0 M). The solid was collected by filtration and then washed with Et_2_O. The solid was identified as Cbz-L-Lys(Cbz)-*Z*-ΔPhe-OH (**3**) (0.749 g, 99%). ^1^H NMR (400 MHz, DMSO-d_6_) δ: 1.24–1.47 (4H, m, γ-CH_2_ and δ-CH_2_ of Lys), 1.51–1.78 (2H, m, β-CH_2_ of Lys), 2.91–3.03 (2H, m, ε-CH_2_ of Lys), 4.05-4.14 (1H, m, α-CH of Lys), 4.99 (2H, s, 1 × CH_2_ of Cbz), 5.05 (2H, s, 1 × CH_2_ of Cbz), 7.17–7.39 (15H, m, ArH and β-CH of ΔPhe and NH), 7.43-7.61 (3H, m, ArH and NH), 9.43 (1H, s, NH of ΔPhe). ^13^C NMR (100.6 MHz, DMSO-d_6_, δ): 22.7 (CH_2_, γ-CH_2_ of Lys), 29.0 (CH_2_, δ-CH_2_ of Lys), 31.2 (CH_2_, β-CH_2_ of Lys), 40.1 (CH_2_, ε-CH_2_ of Lys), 54.9 (CH, α-CH of Lys), 65.1 (CH_2_, 1 × CH_2_ of Cbz), 65.4 (CH_2_, 1 × CH_2_ of Cbz), 126.3 (CH, Ar), 126.5 (CH, Ar), 127.4 (C, Ar), 127.7 (CH, Ar), 127.8 (CH, Ar), 128.0 (CH, Ar), 128.2 (CH, Ar), 128.3 (CH, Ar), 128.7 (CH, Ar), 129.1 (CH, Ar), 129.8 (CH, β-CH of ΔPhe), 134.1 (C, α-C of ΔPhe), 137.0 (C, 2 × C of Cbz), 156.0 (C, 2 × C=O of Cbz), 166.3 (C, C=O), 171.3 (C, C=O). HRMS (ESI) *m*/*z*: [M + H]^+^ calcd for C_31_H_34_N_3_O_7_ 560.2397; found: 560.2397.

### 4.2. Hydrogel Preparation and Determination of Critical Gelation Concentration (CGC)

NaOH (1.0 M) was added to a mixture of the hydrogelators **1**, **2**, or **3** (0.3, 0.4, 0.5, 0.6, and 0.7 mg) in water (1.0 mL) in a small glass vial until pH 10 was reached (~30 μL). The resulting mixture was sonicated for ~1 min until dissolved to afford gelator concentrations of 0.03, 0.04, 0.05, 0.06, and 0.07 wt%, respectively. D-glucono-d-lactone (GdL) (3.0 mg) was added to each vial, followed by thorough mixing for 1 min. The solutions were then left undisturbed overnight. The CGC was then determined through tube inversion tests (tubes which revealed a free-standing material following 5 min of inversion were deemed to be gels). From these tests, it was observed that hydrogelator **1** had a CGC of 0.05 wt% and hydrogelator **2** had a CGC of 0.07 wt%. Hydrogelator **3** required a higher concentration to form a hydrogel, and therefore the above procedure was repeated with increased amounts of **3** (1.7, 1.8, 1.9, 2.0, 2.1, and 2.2 mg), which corresponded to gelator concentrations of 0.17, 0.18, 0.19, 0.20, 0.21, and 0.22 wt%. The CGC of **3** was determined to be 0.20 wt%.

### 4.3. Scanning Transmission Electron Microscopy (STEM)

STEM images were recorded using a NanoSEM—FEI Nova 200 (FEI Technologies, Inc., Hillsboro, OR, USA), operating at 15 kV, coupled to an Electron Dispersive Spectroscopic analyser (EDS) and Electron Backscatter Diffraction EDAX—Pegasus X4M analyser and detection system (EBSD) at SEMAT (Serviços de Caracterização de Materiais), Guimarães, Portugal. After preparation of the hydrogel, a small portion of each sample was placed onto a TEM 400 mesh copper grid with Formvar/Carbon, held by tweezers, and the excess solution was cleaned. The processing of STEM images was performed using ImageJ software (National Institutes of Health (NIH), Bethesda, MD, USA), which consisted of enhancing local contrast and adjusting brightness followed by manual selection of fibres.

### 4.4. Circular Dichroísm Spectroscopy

Circular dichroism spectra were recorded under a constant flow of N_2_ using a spectropolarimeter Jasco model J-1500 (JASCO, Tokyo, Japan) at 25 °C using solutions of hydrogelators **1**, **2**, and **3** (0.01 wt%). The solutions of the hydrogelators were loaded into 0.1 mm quartz cells.

### 4.5. Rheological Studies

Gel-forming solutions of compounds **1**, **2**, and **3** were injected into the Couette cell (with 1 mL volume and 0.5 mm gap) of a stress-controlled rotational rheometer (MCR300, Anton Paar GmbH, Graz, Austria). All the viscoelastic characterisation was performed at 25 °C using the temperature control of the Couette cell. The sample homogenisation was achieved by applying a shear rate of 5 s^−1^ for one minute immediately after sample loading into the shear cell. Then, a small amplitude oscillatory shear (SAOS) with a frequency of 1 Hz and an amplitude of 0.01% was applied during 10 h, whereas both shear storage (G’) and loss (G”) moduli were recorded every 100 *s* to monitor the gel kinetics. The gels’ mechanical spectra were then measured by performing a frequency sweep (from 100 to 0.01 Hz), using the same SAOS amplitude as during the gel kinetics. This was followed by a dynamic strain sweep performed at 1 Hz up to 100% to test for gel break-up.

### 4.6. Sustained Release Assays

Hydrogels of **1** were prepared to form 1 mL hydrogels containing the same concentration of the hydrogelators described above and the appropriate cargo—methylene blue (0.1 nM), methyl orange (0.2 nM), or ciprofloxacin (0.2 nM)—in a slightly modified version of the procedure described by Abraham et al. [30]. After allowing to stand overnight, 1 mL of water was carefully added to the surface of the hydrogels. Aliquots of the layered solution (100 μL) were removed at 1 h, 2 h, 3 h, 4 h, 6 h, 24 h, 72 h, and 6 days from the time the water was initially layered on top of the hydrogel. After removing each aliquot, the volume water was immediately replaced by an equal volume of water. The concentration of methylene blue or methyl orange in each aliquot was determined by measuring the absorbance at λmax of the dye (666 nm for methylene blue and 465 nm for methyl orange) using a microplate reader and then converting the value to percentage release (using a standard calibration curve). The concentration of ciprofloxacin in each aliquot was determined using analytical HPLC, where the integrated peak area was converted to a percentage release (using a standard calibration curve). Each experiment was performed in triplicate, and the mean percentage cargo release was plotted against time.

### 4.7. Cell Culture

Human keratinocytes cell line HaCaT was from ATCC. Cells were cultured in DMEM, supplemented with 10% FBS and 1% penicillin/streptomycin, and were incubated in an incubator at 37 °C in a humidified atmosphere of 5% CO_2_.

### 4.8. MTT Assay

Cells were seeded in 96-well plates (1.5 × 10^4^ cells/well) and left to attach for 24 h. After this period, cells were incubated with different concentrations of the molecules under study for another 24 h. After this period, cell viability was evaluated based on the ability of metabolically active cells to convert MTT to formazan over the course of 2 h. Absorbances were measured at 570 nm in a Multiskan GO plate reader (Thermo Fisher Scientific; Waltham, MA, USA) and results were expressed as percentage of the respective control and correspond to the mean ± standard error of the mean (SEM) of at least three independent experiments performed in triplicate.

### 4.9. LDH Leakage

Cells were seeded in 96-well plates (1.5 *×* 10*^4^* cells per well) and left to attach for 24 h. After this period, cells were incubated with different concentrations of the molecules under study for another 24 h. To assess the release of the stable cytosolic enzyme lactate dehydrogenase (LDH) into the media, 24 h after the incubation of the cells with the different concentrations of the molecules under study, 40 µL of culture media was removed to a 96-well plate. Triton X-100 (1%) was used as a positive control to assure cell lysis (30 min). LDH released was determined using a CytoTox 96*^®^* assay kit (Promega; Madison, WI, USA) according to the manufacturer’s instructions. Absorbances were measured at 490 nm in a Multiskan GO plate reader (Thermo Fisher Scientific; Waltham, MA, USA), and results correspond to the fold-increase of absorbance in treated vs. untreated cells of three independent experiments performed in duplicate. Following assessment of the distribution of the results, ANOVA was performed using GraphPad Prism 8.0 (GraphPad Prism Inc., San Diego, CA, USA).

### 4.10. Computer Modelling

The dehydrodipeptides **1**–**3** were modelled to unveil their preferable conformation and interaction pattern. The molecular structure of each peptide was designed with PyMOL [35] and then submitted to the ATB server [36] for structural optimisation and generation of GROMOS 54a7 force field parameters [37]. Molecular dynamics simulations were conducted in cubic boxes solvated with the SPC water model [38]. For simplicity, the C-terminal amino acid was built in its neutral form, to despise the effect of ions on peptides interactions. Two types of systems were addressed: the first one with only one unit of the peptide centred in the box and the second where two units are apart from each other. The average volume of the boxes was of 52 nm^3^. In both cases, infinite dilution conditions were considered. Each system was subjected to energy minimisation with the steepest descent algorithm. NVT and NPT initialisation steps were conducted for 100ps each. Next, 60 ns of MD simulations were run. The GROMACS 5.1.4 software package [39] was used. Temperature and pressure were maintained constant at 300K and 1 atm, respectively, by using the V-rescale thermostat [40] and the Parrinello–Rahman barostat [41]. The LINCS [42] algorithm was used to constrain bond lengths and angles and SETTLE [43] in the case of water molecules, which allowed the use of a 2 fs time step. Electrostatics and van de Waals interactions were treated using the PME method [44] and the Verlet cutoff scheme.

To analyse the monomers in solution, the backbone RMSD was tracked, and the central structure was determined using a clustering algorithm and a single-linkage method. The dimers’ aggregation process was followed by measuring the distance between the units’ COM over time. Additionally, the radial distribution functions (RDFs) were computed to infer the probability of interaction at a certain distance.

## Figures and Tables

**Table 1:** Critical gelation concentration and pH of dehydrodipeptides **1**–**3**.

Peptide	Critical Gelation Concentration (CGC)		pH	cLogP ^1^
	wt%	mM		
1	0.05	0.80	4.8	6.16
2	0.07	1.18	5.1	5.39
3	0.20	3.57	5.0	6.42

^1^ cLogP values obtained from https//molinspiration.com, accessed on 9 May 2022.

**Table 2:** G’ and G’’ for hydrogels **1**–**3** at 0.20 wt%.

Hydrogel	G’ (Pa)	G″ (Pa)
**1**	3.63 × 10^3^	221
**2**	1.83 × 10^3^	145
**3**	1.84 × 10^2^	28

## Data Availability

Not applicable.

## Supplementary Materials

The following supporting information can be downloaded at https://www.mdpi.com/article/10.3390/ijms231911811/s1: Experimental procedures and characterisation data for compounds **4**, **5a**, **6a**–**c**, **7a**-**c**, **8** and **9**.
