# Supplementary material for: Aryl-Capped Lysine-Dehydroamino Acid Dipeptide Supergelators as Potential Drug Release Systems
Source: Int J Mol Sci. 2022 Oct 5;23(19):11811. doi: 10.3390/ijms231911811 (PMC9569917; doi:10.3390/ijms231911811)
Supplement: Supplementary file 1 [file ijms-23-11811-s001.zip › ijms-1947441-supplementary.pdf]

Supporting Information

Aryl-Capped Lysine-Dehydroamino Acid Dipeptide Supergelators as Potential Drug Release Systems

Carlos Oliveira [1], Renato B. Pereira [2], David M. Pereira [2], Loic Hilliou [3], Tarsila G. Castro [4,5], José A. Martins [1*], Peter J. Jervis [1,*] and Paula M.T. Ferreira [1]

Experimental procedures and characterisation data for compounds **4**, **5a**, **6a–c**, **7a-c**, **8** and **9**:

Synthesis of H-D,L-Phe(β-OH)-OMe•HCl (**4**):

β-hydroxyphenylalanine monohydrate [H-D,L-Phe(β-OH)-OH•H_2O] (10 mmol, 1.80 g) was added to MeOH (40 mL) at 0 °C . With stirring, thionyl chloride 0.80 mL, 1.1 equiv) was added dropwise over 10 min and then the reaction mixture was allowed to warm to room temperature, before being heated at 40 °C for 4 h. The solvent was removed under reduced pressure and Et_2O was added and then removed under pressure. This process was repeated until a white solid of compound H-D,L-Phe(β-OH)-OMe•HCl (**4**) was formed (85%, 2.02 g). ^{1}H NMR (400 MHz, DMSO-d_6) δ: 3.57 (3H, s, OC*H*$_3$); 4.11 (1H, br q, β-C*H*), 5.00 (1H, d, J 5.6 Hz, α-C*H*), 6.56 (1H, br s, O*H*), 7.31–7.38 (5H, m, Ar*H*), 8.56 (3H, s, *H*$_3$N$^+$).

Synthesis of Boc-L-Lys(Boc)-OH (**5a**):

L-Lysine monohydrate (2.00g, 11.0 mmol) was dissolved in 1,4-dioxane (65 mL) and then a solution of NaOH 1.0 M (32.7 mL, 33.0 mmol) was added. The solution was left to stir for 10 minutes before Boc_2O (2.00 equiv, 4.5 g, 22.0 mmol) was added. The mixture was then stirred at rt overnight. The 1,4-dioxane solvent was removed under reduced pressure and the mixture was acidified with $KHSO_4$ (1.0 M) until pH 2–3. The aqueous phase was extracted with ethyl acetate (3 x 50 mL) and the organic phase dried with $MgSO_4$. Removal of the solvent under reduced pressure afforded Boc-L-Lys(Boc)-OH (**5a**) as a transparent oil (2.27 g, 60%). ^{1}H NMR (400 MHz, DMSO-d_6) δ: 1.19–1.40 (4H, m, γ-C*H*$_2$ and δ-C*H*$_2$), 1.42–1.69 (2H, m, β-C*H*$_2$), 2.81–2.90 (2H, m, ε-C*H*$_2$), 3.77–3.87 (1H, m, α-C*H*), 6.74 (1H, t, J 18.0 Hz, δ-N*H*), 6.92 (1H, d, J 8.0 Hz, α-C*H*), 12.37 (1H, br s, CO_2*H* of Lys).

Synthesis of Boc-L-Lys(Boc)-D,L-Phe(β-OH)-OMe (**6a**):

Boc-L-Lys(Boc)-OH (**5a**) (0.500 g, 1.50 mmol) was dissolved in MeCN (8 mL) and cooled to 0 °C. HBTU (1.1 equiv, 0.630 g, 1.65 mmol), H-D,L-Phe(β-OH)-OMe•HCl (**4**) (1.00 equiv, 0.349 g, 1.50 mmol) and triethylamine (3 equiv, 0.7 mL, 4.50 mmol) were added sequentially, with 2 min between each addition. The mixture was stirred at rt overnight. The solvent was removed under reduced pressure to afford a residue that was partitioned between EtOAc (50 mL) and $KHSO_4$ (1.0 M, 50 mL). After separation of the phases, the organic layer was thoroughly washed with $KHSO_4$ (1.0 M, 2 x 50 mL), $NaHCO_3$ (1.0 M, 2 x 50 mL) and brine (2 x 50 mL) and then dried with $MgSO_4$. Filtration followed by removal of the solvent under reduced pressure afforded a diastereomeric mixture of Boc-L-Lys(Boc)- D,L-Phe(β-OH)-OMe (**6a**) as a white solid (0.557 g, 71%). ^{1}H NMR (400 MHz, DMSO-d_6) δ: 0.90–1.11 (4H, m, γ-C*H*$_2$ and δ-C*H*$_2$ of Lys), 1.16–1.29 (2H, m, β-C*H*$_2$ of Lys), 1.36 (9H, s, 1 x OC(C*H*$_3$)$_3$), 1.38 (9H, s, 1 x OC(C*H*$_3$)$_3$)], 2.76–2.87 (2H, m, ε-C*H*$_2$ of Lys), 3.60 and 3.64 (3H, s, OC*H*$_3$), 3.76–3.85 and 3.88–3.94 (1H, m, α-C*H* of Phe), 4.53 (1H, dd, J 8.8 Hz, 2.8 Hz, α-C*H* of Lys), 5.09–5.15 (1H, m, β-C*H* of Phe), 5.89-5.97 (1H, m, 1 x N*H*), 6.91 (1H, d, J 8.4 Hz, 1 x N*H*), 7.19–7.39 (5H, m, Ar*H*), 7.45 and 7.90 (1H, d, J 8.8 Hz, 1 x N*H*).

Synthesis of Boc-L-Lys(Boc)-*Z*-ΔPhe-OMe (**7a**):

DMAP (0.11 equiv, 0.014 g, 0.12 mmol) and Boc_2O (1,1 equiv, 0.262 g, 1.20 mmol) were added to a solution of Boc-L-Lys(Boc)-D,L-Phe(β-OH)-OMe (**6a**) (0.5566 g, 1.09 mmol) in dry MeCN (8 mL) under rapid stirring at rt. The mixture was monitored by ^{1}H NMR and stirred at rt until all the starting material was consumed (typically 5 h). *N,N,N′,N′*-tetramethylguadinine (4% in volume, 0.32 mL) was added. The mixture was stirred at rt and monitored by ^{1}H NMR until all the intermediate was consumed. Concentration under reduced pressure gave a residue that was partitioned between EtOAc (50 mL) and $KHSO_4$ (1.0 M, 30 mL). After separation of the phases, the organic phase was washed with $KHSO_4$ (1.0 M, 2 x 60 mL), $NaHCO_3$ (1.0 M, 2 x 60 mL) and brine (2 x 60 mL) and then dried with $MgSO_4$. Removal of the solvent afforded Boc-L-Lys(Boc)-*Z*-ΔPhe-OMe (**7a**) (0.363 g, 66%). ^{1}H NMR (400 MHz, DMSO-d_6) δ: 1.25–1.42 (4H, m, γ-C*H*$_2$ and δ-C*H*$_2$ of Lys), 1.36 (9H, s, OC(C*H*$_3$)$_3$), 1.40 (9H, s, OC(C*H*$_3$)$_3$), 1.49–1.71 (2H, m, β-C*H*$_2$ of Lys), 2.83–2.95 (2H, m, ε-C*H*$_2$ of Lys), 3.68 (3H, s, OC*H*$_3$), 4.00 (1H, dd, J 14.4 Hz, 7.2 Hz, α-C*H* of Lys), 6.75 (1H, s, N*H*), 6.92 (1H, d, J 7.6 Hz, 1 x N*H*), 7.22 (1H, s, β-C*H* of ΔPhe); 7.33–7.41 (3H, m, Ar*H*); 7.65–7.74 (2H, m, Ar*H*), 9.59 (1H, s, N*H* of ΔPhe).

Synthesis of H-L-Lys-*Z*-ΔPhe-OMe•2TFA:

Boc-L-Lys(Boc)-*Z*-ΔPhe-OMe (**7a**) (0.363 g, 0.72 mmol) was dissolved in TFA (3.0 mL) and the reaction mixture was stirred at room temperature for 1 h. The TFA was removed under reduced pressure. Traces of residual TFA were removed by the addition of CH_2Cl_2 (3 x 10 mL) followed by removal under reduced pressure, affording H-L-Lys-*Z*-ΔPhe-OMe•2TFA as a brown oil. ^{1}H NMR (400 MHz, DMSO-d_6) δ: 1.31–1.60 (4H, m, γ-C*H*$_2$ and δ-C*H*$_2$ of Lys), 1.73–1.88 (2H, m, β-C*H*$_2$ of Lys), 2.69–2.80 (2H, m, ε-C*H*$_2$ of Lys), 3.13 (3H, s, OC*H*$_3$), 3.95–4.06 (1H, m, α-C*H* of Lys), 7.36–7.47 (4H, m, Ar*H* and β-C*H* of ΔPhe), 7.63–7.69 (2H, m, Ar*H*), 7.74 (1H, br s, N*H*), 8.24 (3H, d, J 3.6 Hz, *H*$_3$N$^+$ of Lys), 10.20 (1H, s, *H*$_3$N$^+$ of ΔPhe).

Naph-L-Lys(Naph)-*Z*-ΔPhe-OMe (**8**):

H-L-Lys-*Z*-ΔPhe-OMe•2TFA (0.3826g, 0.72 mmol) was dissolved in MeCN (8 mL) and cooled to 0 °C. HBTU (1.1 equiv, 0.300 g, 0.79 mmol), 2-naphthylacetic acid (2.10 equiv, 0.281 g, 1.51 mmol) and triethylamine (3 equiv, 0.3 mL, 2.15 mmol) were added sequentially, with 2 min between each addition, and then the mixture was stirred at rt overnight. The solvent was removed under reduced pressure to afford a residue that was partitioned between EtOAc (50 mL) and $KHSO_4$ (1.0 M, 50 mL). After separation of the phases, the organic layer was thoroughly washed with $KHSO_4$ (1.0 M, 2 x 50 mL), $NaHCO_3$ (1.0 M, 2 x 50 mL) and brine (2 x 50 mL) and then dried with $MgSO_4$. Filtration followed by removal of the solvent under reduced pressure afforded Naph-L-Lys(Naph)-*Z*-ΔPhe-OMe (**8**) as a white solid (0.400 g, 87%). ^{1}H NMR (400 MHz, DMSO-d_6) δ: 1.27–1.48 (4H, m, γ-C*H*$_2$ and δ-C*H*$_2$ of Lys), 1.57–1.81 (2H, m, β-C*H*$_2$ of Lys), 2.96–3.09 (2H, m, ε-C*H*$_2$ of Lys), 3.55 (3H, s, OC*H*$_3$ of ΔPhe), 3.66 (4H, s, 2 x C*H*$_2$ of Naph), 4.33–4.45 (1H, m, α-C*H*$_2$ of Lys), 7.18–7.49 (10H, m, Ar*H* and β-C*H* of ΔPhe), 7.60–7.89 (10H, m, Ar*H* and N*H*), 8.06–8.13 (1H, m, Ar*H*), 8.40 (1H, d, J 8.4, 1 x N*H*), 9.76 (1H, s, 1 x N*H* of ΔPhe). ^{13}C NMR (100.6 MHz, DMSO-d_6, δ): 22.6 (CH_2, γ-CH_2 of Lys), 28.7 (CH_2, δ-CH_2 of Lys), 31.2 (CH_2, β-CH_2 of Lys), 38.5 (CH_2, ε-CH_2 of Lys), 42.0 (CH_2, 1 x CH_2 of Naph), 42.5 (CH_2, 1 x CH_2 of Naph), 52.1 (CH_3, OCH_3 of ΔPhe), 52.7 (CH, α-CH of Lys), 125.4 (CH, Ar), 126.0 (CH, Ar), 127.2 (CH, Ar), 127.30 (CH, Ar), 127.35 (CH, Ar), 127.4 (CH, Ar), 127.50 (CH, Ar), 127.55 (CH, Ar), 127.63 (CH, Ar), 127.67 (CH, Ar), 127.7 (CH, Ar), 127.8 (CH, Ar), 128.3 (CH, Ar), 128.4 (CH, Ar), 129.3 (CH, Ar), 129.9 (CH, Ar), 130.0 (CH, Ar), 131.7 (C, Ar), 131.9 (C, Ar), 132.0 (C, Ar), 132.1 (CH, β-CH of ΔPhe), 132.93 (C, Ar), 132.97 (C, Ar), 133.1 (C, α-C of ΔPhe), 134.1 (C, Ar), 134.2 (C, Ar), 165.3 (C, C=O), 169.9 (C, C=O of Naph), 170.2 (C, C=O of Naph), 171.9 (C, C=O).

Synthesis of Boc-L-Lys(Cbz)-D,L-Phe(β-OH)-OMe (**6b**):

Boc-L-Lys(Cbz)-OH (0.800 g, 2.15 mmol) was dissolved in MeCN (5 mL) and cooled to 0 °C. HBTU (1.1 equiv, 0.900 g, 2.37 mmol), H-D,L-Phe(β-OH)-OMe (**4**) 1.00 equiv, 0.500 g, 2.15 mmol) and triethylamine (3 equiv, 0.9 mL, 6.47 mmol) were added sequentially, with 2 min between each addition, and then the mixture was stirred at rt overnight. The solvent was removed under reduced pressure to afford a

residue that was partitioned between EtOAc (50 mL) and $KHSO_4$ (1.0 M, 50 mL). After separation of the phases, the organic layer was thoroughly washed with $KHSO_4$ (1.0 M, 2 x 50 mL), $NaHCO_3$ (1.0 M, 2 x 50 mL) and brine (2 x 50 mL) and then dried with $MgSO_4$. Filtration followed by removal of the solvent under reduced pressure afforded a diastereomeric mixture of Boc-L-Lys(Cbz)-D,L-Phe(β-OH)-OMe (**6b**) as a white solid (1.146 g, 96%). ^{1}H NMR (400 MHz, DMSO-d_6) δ: 0.91–1.04 (4H, m, γ-CH_2 and δ-CH_2 of Lys), 1.29–1.43 (2H, m, β-CH_2 of Lys), 1.36 and 1.37 (9H, s, OC(CH_3)$_3$), 2.81–2.95 (2H, m, ε-CH_2 of Lys), 3.60 and 3.64 (3H, s, OCH_3), 3.77–3.89 and 3.88–3.97 [1H, m, α-C*H* of Phe(β-OH)], 4.54 (1H, dd, J 9.2 Hz, and 2.8 Hz, α-C*H* of Lys), 4.99 (2H, s, CH_2 of Cbz), 5.09–5.18 (1H, m, β-C*H* of Phe(β-OH)), 5.88–5.95 (1H, m, 1 x N*H*), [6.72 and 6.96 (1H, d, J 8.4 Hz, 1 x N*H*)], 7.10–7.39 (10H, m, Ar*H*), 7.74 and 7.91 (1H, 2 d, J 8.8 Hz, 1 x N*H*).

Synthesis of Boc-L-Lys(Cbz)-Z-ΔPhe-OMe (**7b**):

DMAP (0.11 equiv, 0.026 g, 0.23 mmol) and Boc_2O (1.1 equiv, 0.493 g, 2.26 mmol) were added to a solution of Boc-L-Lys(Cbz)-D,L-Phe(β-OH)-OMe•HCl (**4**) (1.1459 g, 2.05 mmol) in MeCN (10 mL) under rapid stirring at rt. The mixture was monitored by ^{1}H NMR and stirred at rt until all the starting material was consumed (typically 5 h). *N,N,N′,N′*-tetramethylguadinine (4% in volume, 0.4 mL) was added. The mixture was stirred at rt and monitored by ^{1}H NMR until all the intermediate was consumed. Concentration under reduced pressure gave a residue that was partitioned between EtOAc (50 mL) and $KHSO_4$ (1.0 M, 30 mL). After separation of the phases, the organic phase was washed with $KHSO_4$ (1.0 M, 2 x 60 mL), $NaHCO_3$ (1.0 M, 2 x 60 mL) and brine (2 x 60 mL) and then dried with $MgSO_4$. Removal of the solvent afforded Boc-L-Lys(Cbz)-Z-ΔPhe-OMe (**7b**) (0.860 g, 78%). ^{1}H NMR (400 MHz, DMSO-d_6) δ: 1.25–1.43 (4H, m, γ-CH_2 and δ-CH_2 of Lys), 1.40 (9H, s, OC(CH_3)$_3$), 1.49–1.71 (2H, m, β-CH_2 of Lys), 2.91–3.02 (2H, m, ε-CH_2 of Lys), 3.67 (3H, s, OCH_3 of ΔPhe), 3.96–4.09 (1H, m, α-C*H* of Lys), 4.99 (2H, s, CH_2 of Cbz), 6.90–6.98 (1H, d, J 7.6 Hz, N*H*), 7.19–7.39 (10H, m, Ar*H* and β-C*H* of ΔPhe and 1 N*H*), 7.67–7.75 (2H, m, Ar*H*), 9.59 (1H, s, N*H* of ΔPhe).

Synthesis of H-L-Lys(Cbz)-Z-ΔPhe-OMe•TFA:

Boc-L-Lys(Cbz)-Z-ΔPhe-OMe (**7b**) (0.358 g, 0.88 mmol) was dissolved in TFA (3.0 mL) and the reaction mixture was stirred at room temperature for 1 hour. The TFA was then removed under reduced pressure. Traces of residue TFA were removed by the addition of CH_2Cl_2 (3 x 10 mL) followed by removal under reduced pressure, affording H-L-Lys(Cbz)-Z-ΔPhe-OMe•TFA as a brown oil. ^{1}H NMR (400 MHz, DMSO-d_6) δ: 1.28–1.49 (4H, m, γ-CH_2 and δ-CH_2 of Lys), 1.71–1.89 (2H, m, β-CH_2 of Lys), 2.91–3.04 (2H, m, ε-CH_2 of Lys), 3.73 (3H, s, OCH_3 of ΔPhe), 3.90–4.08 (1H, m, α-C*H* of Lys), 4.99 (2H, s, CH_2 of Cbz), 7.28 (1H, t, J 5.6 Hz, 1 x N*H*), 7.10–7.46 (9H, m, Ar*H*), 7.62–7.68 (2H, m, Ar*H*), 8.21 (3H, d, J 3.6 Hz, H_3N^+); 10.19 (1H, s, N*H* of ΔPhe).

Synthesis of Naph-L-Lys(Cbz)-Z-ΔPhe-OMe (**9**):

H-L-Lys(Cbz)-Z-ΔPhe-OMe•TFA (0.5374g, 0.98 mmol) was dissolved in MeCN (8 mL) and cooled to 0 °C. 2-Naphthylacetic acid (1.00 equiv, 0.182 g, 0.98 mmol), triethylamine (3 equiv, 0.4 mL, 2.94 mmol) and HBTU (1.1 equiv, 0.409 g, 1.08 mmol) were added sequentially, with 2 min between each addition, and then the mixture was stirred at rt overnight. The solvent was removed under reduced pressure to afford a residue that was partitioned between EtOAc (50 mL) and $KHSO_4$ (1.0 M, 50 mL). After separation of the phases, the organic layer was thoroughly washed with $KHSO_4$ (1.0 M, 2 x 50 mL), $NaHCO_3$ (1.0 M, 2 x 50 mL) and brine (2 x 50 mL) and then dried with $MgSO_4$. Filtration followed by removal of the solvent under reduced pressure afforded Naph-L-Lys(Cbz)-Z-ΔPhe-OMe (**9**) as a white solid (0.572 g, 98%). ^{1}H NMR (400 MHz, DMSO-d_6) δ: 1.22–1.48 (4H, m, γ-CH_2 and δ-CH_2 of Lys), 1.54–1.79 (2H, m, β-CH_2 of Lys), 2.91–3.01 (2H, m, ε-CH_2 of Lys), 3.67 (3H, s, OCH_3 of ΔPhe), 3.68 (2H, s, CH_2 of Naph), 4.37–4.43 (1H, m, α-C*H* of Lys), 4.99 (2H, s, CH_2 of Cbz), 7.19-7.88 (19H, m, Ar*H* and β-C*H* of ΔPhe and 1x N*H*), 8.39 (1H, d, J 7.6 Hz, N*H*), 9.74 (1H, s, N*H* of ΔPhe). ^{13}C NMR (100.6 MHz, DMSO-d_6, δ): 22.6 (CH_2, γ-CH_2 of Lys), 29.1 (CH_2, δ-CH_2 of Lys), 31.3 (CH_2, β-CH_2 of Lys), 40.2 (CH_2, ε-CH_2 of Lys), 42.1 (CH_2, CH_2 of Naph), 48.6 (CH_3, OCH_3 of ΔPhe), 52.7 (CH, α-CH of Lys), 65.1 (CH_2, CH_2 of Cbz), 125.5 (CH, Ar), 126.0 (CH, Ar), 127.33 (CH, Ar), 127.39 (CH, Ar), 127.50 (CH, Ar), 127.56 (CH, Ar),

127.7 (CH, Ar), 128.4 (CH, Ar), 128.5 (CH, Ar), 129.1 (CH, Ar), 129.4 (CH, Ar), 130.0 (CH, Ar), 130.1 (CH, Ar), 131.9 (CH, β-CH of ΔPhe), 139.1 (C, Ar), 132.9 (C, *α*-C of ΔPhe), 133.1 (CH, Ar), 133.4 (C, Ar), 134.1 (C, Ar), 137.2 (C, Ar), 156.0 (C, C=O), 166.1 (C, C=O), 170.2 (C, C=O), 171.5 (C, C=O).

4.1.13. Synthesis of Cbz-L-Lys(Cbz)-Phe(β-OH)-OMe (**6c**):

Cbz-L-Lys(Cbz)-OH (1.78 g, 4.30 mmol) was dissolved in MeCN (10 mL) and cooled to 0 °C. HBTU (1.1 equiv, 1.79 g, 4.73 mmol), H-Phe-β-OH)-OMe•HCl (**4**) (1.00 equiv, 1.00 g, 4.30 mmol) and triethylamine (3 equiv, 1.80 mL, 12.90 mmol) were added sequentially, with 2 min between each addition, and then the mixture was stirred at rt overnight. The solvent was removed under reduced pressure to afford a residue that was partitioned between EtOAc (50 mL) and $KHSO_4$ (1.0 M, 50 mL). After separation of the phases, the organic layer was thoroughly washed with $KHSO_4$ (1.0 M, 2 x 50 mL), $NaHCO_3$ (1.0 M, 2 x 50 mL) and brine (2 x 50 mL) and then dried with $MgSO_4$. Filtration followed by removal of the solvent under reduced pressure afforded a diastereomeric mixture of Cbz-L-Lys(Cbz)-Phe(β-OH)-OMe (**6c**) as a white solid (1.82 g, 71%). ^{1}H NMR (400 MHz, DMSO-d_6) δ: 0.98–1.17 (4H, m, γ-C*H*$_2$ and δ-C*H*$_2$ of Lys), 1.30–1.42 (2H, m, β-C*H*$_2$ of Lys), 2.81–2.96 (2H, m, ε-C*H*$_2$ of Lys), 3.59 and 3.64 (3H, s, OC*H*$_3$), 3.91–4.08 (1H, m, *α*-C*H* of Phe), 4.49–4.60 (1H, m, *α*-C*H* of Lys), 5.09 and 5.15 (4H, s, 2 x C*H*$_2$ of Cbz), 5.07–5.19 (1H, m, β-C*H* of Phe), 5.89–5.97 (1H, m, N*H*), 7.14–7.39 (16H, m, Ar*H* and N*H*), 7.89 and 8.02 (1H, 2 d, J 9.2 Hz, N*H*)].

Synthesis of Cbz-L-Lys(Cbz)-*Z*-ΔPhe-OMe (**7c**):

DMAP (0.11 equiv, 0.025 g, 0.21 mmol) and Boc_2O (1,1 equiv, 0.451 g, 2.07 mmol) were added to a solution of Cbz-L-Lys(Cbz)-Phe(β-OH)-OMe (**6c**) (1.114 g, 1.88 mmol) in dry MeCN (8 mL) under rapid stirring at rt. The mixture was monitored by ^{1}H NMR and stirred at rt until all the starting material was consumed (typically 5 h). *N*,*N*,*N'*,*N'*-tetramethylguadinine (4% in volume, 0.32 mL) was added. The mixture was stirred at rt and monitored by ^{1}H NMR until all the intermediate was consumed. Concentration under reduced pressure gave a residue that was partitioned between EtOAc (50 mL) and $KHSO_4$ (1.0 M, 30 mL). After separation of the phases, the organic phase was washed with $KHSO_4$ (1.0 M, 2 x 60 mL), $NaHCO_3$ (1.0 M, 2 x 60 mL) and brine (2 x 60 mL) and then dried with $MgSO_4$. Filtration followed by removal of the solvent afforded Cbz-L-Lys(Cbz)-*Z*-ΔPhe-OMe (**7c**) (0.785 g, 73%). ^{1}H NMR (400 MHz, DMSO-d_6) δ: 1.28–1.47 (4H, m, γ-C*H*$_2$ and δ-C*H*$_2$ of Lys), 1.52–1.77 (2H, m, β-C*H*$_2$ of Lys), 2.90–3.00 (2H, m, ε-C*H*$_2$ of Lys), 3.67 (3H, s, OC*H*$_3$), 4.01 (1H, dd, J 37.6 Hz, 6.0 Hz, *α*- C*H* of Lys), 4.99 (2H, s, 1 x C*H*$_2$ of Cbz), 5.05 (2H, s, 1x C*H*$_2$ of Cbz), 7.17–7.40 (15H, m, Ar*H* and β-C*H* of ΔPhe and 1 x N*H*), 7.46 (1H, d, J 7.6 Hz, 1 x N*H*), 7.64–7.71 (2H, m, Ar*H*), 9.67 (1H, s, N*H* of ΔPhe). ^{13}C NMR (100.6 MHz, DMSO-d_6, δ): 22.6 (CH_2, γ-CH_2 of Lys), 29.0 (CH_2, δ-CH_2 of Lys), 30.9 (CH_2, β-CH_2 of Lys), 40.1 (CH_2, ε-CH_2 of Lys), 52.8 (CH_3, OCH_3 of ΔPhe), 54.7 (CH, *α*-CH of Lys), 65.2 (CH_2, 1 x CH_2 of Cbz), 65.4 (CH_2, 1 x CH_2 of Cbz), 126.0 (C, Ar), 127.72 (CH, Ar), 127.75 (CH, Ar), 127.8 (CH, Ar), 128.3 (CH, Ar), 128.5 (CH, Ar), 129.4 (CH, Ar), 129.5 (CH, Ar), 130.0 (CH, Ar), 130.1 (CH, Ar), 132.0 (CH, β-CH of ΔPhe), 133.2 (C, *α*-C of ΔPhe), 137.0 (C, Ar), 137.2 (C, Ar), 156.1 (C, 2 x C=O of Cbz), 165.3 (C, C=O), 172.3 (C, C=O).
